# Orbital septum attachment site on the levator aponeurosis sling for mild congenital blepharoptosis

**DOI:** 10.1007/s10792-024-02967-4

**Published:** 2024-02-05

**Authors:** Jianwei Yang, Lihua Song, Yan Tan, Lulu Zhang, Juan Wang, Limin Liu

**Affiliations:** Hebei Eye Hospital, Xingtai, Hebei Province China

**Keywords:** Mild congenital blepharoptosis, Attachment site, Orbital septum, Levator aponeurosis, Sling

## Abstract

**Purpose:**

This study aimed to investigate the value of the orbital septum attachment site on the levator aponeurosis (OSASLA) sling in correcting mild congenital blepharoptosis.

**Methods:**

A total of 60 patients (92 eyes) with mild congenital blepharoptosis (levator function ≥ 8 mm) were treated in our hospital from January to October 2021, and relevant data of these patients were collected. All patients underwent OSASLA sling for ptosis correction. The distances from the superior tarsal border to the OSASLA were measured. The primary outcome was the number of postoperative changes in the marginal reflex distance 1 (MRD1). Pearson’s correlation coefficient between the distance from the superior tarsal border to the OSASLA and the height of the upper eyelid elevated was analyzed.

**Results:**

Fifty-eight patients (89 eyes) successfully underwent OSASLA sling surgery. The preoperative MRD1 was 1.4–3.6 mm (mean 2.1 ± 0.5 mm), and the postoperative MRD1 was 3.4–5.0 mm (mean 3.7 ± 0.6 mm). The distance from the superior tarsal border to the OSASLA sling was significantly and positively correlated with the height of the upper eyelid elevation (r = 0.7328, P < 0.0001). The eyelid margin positions of the patients did not regress substantially during 6–18 months of follow-up.

**Conclusions:**

Compared with the shortening of levator palpebrae superioris (LPS) and pleating of LPS, the OSASLA sling is a less invasive, more effective, and easy-operating surgery for mild congenital blepharoptosis.

## Introduction

Congenital blepharoptosis is an autosomal dominant inheritance mainly caused by dysplasia of the oculomotor nuclei [1] or the levator palpebrae superioris (LPS) muscle [2]. The manifestation is incompetence or loss of functions of LPS muscles and Müller's smooth muscles, resulting in partial or complete ptosis. It can cause cosmetic problems, affect visual function, and even induce psychological distress [3]. Various methods are available to treat mild blepharoptosis, but the results are not all satisfactory. During operations, we found that the orbital septum attachment site on the levator aponeurosis (OSASLA) was dense. Therefore, we performed the OSASLA sling to correct mild ptosis and obtained satisfactory results.

## Material

A total of 60 patients (92 eyes) with mild congenital blepharoptosis (LPS muscle strength ≥ 8 mm) [4] were treated from January to October 2021 in our hospital, whose data were collected. Of these patients, 35 were male and 25 were female, aged 11–42 years (mean 20.5 ± 5.5 years). The dominant eye was not examined. All the patients had corrected visual acuity ≥ 0.8, and the cover test was performed to exclude strabismus. No patients had limited eye movement, strabismus, or amblyopia before surgery. The levator function was 8–11 mm (mean 9.3 ± 1.2 mm). The margin reflex distance 1 (MRD1) was 1.4–3.6 mm (mean 2.1 ± 0.4 mm). Seventeen cases were in the right eye, 11 in the left eye, and 32 in both eyes. Fifty-nine patients had no operation before, while one patient had double-eyelid surgery.

## Methods

**Fig. 1:**
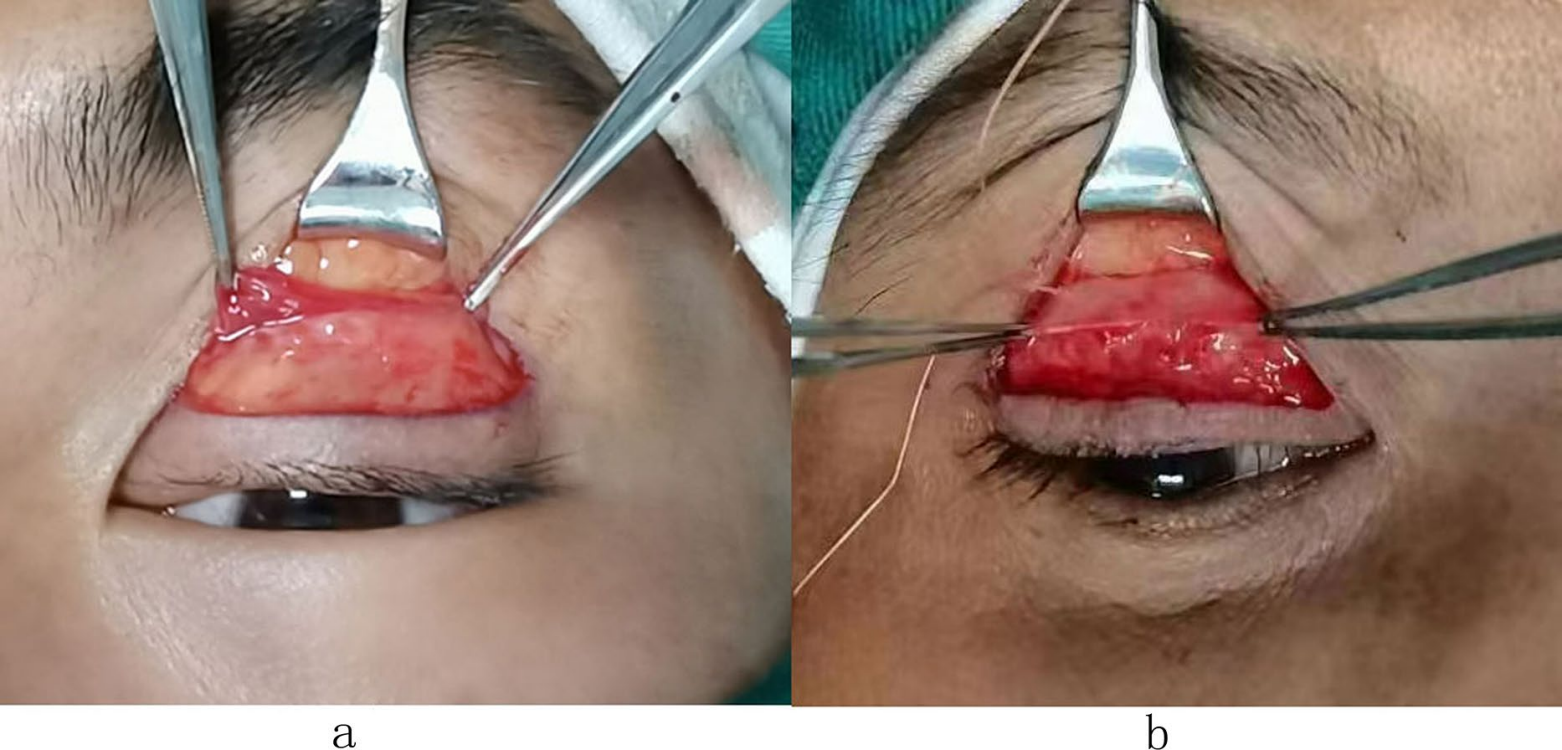
**a** Location of the orbital septum attachment sites on the levator aponeurosis (OSASLA). **b** Sling upper tarsus to the OSASLA

The levator function was considered mild if the lid excursion was 8 mm or greater, moderate if ranging from 5 to 7 mm, and poor if less than 5 mm [5, 6]. We performed all surgeries under local anesthesia and designed the double-eyelid marking lines in the supine position. The width of the double eyelid was approximately 5–7 mm. We lifted the upper eyelid to the proper height with the tips of smooth forceps and marked the double-eyelid line according to the curvature of the upper eyelid crease. The skin and orbicularis oculi muscle were cut open along the marked line. We removed a part of the orbicularis oculi muscle in front of the tarsus near the margin. Following cutting open the orbital septum horizontally, the attachment site of the orbital septum on the LA was exposed (Fig. 1a). The distances from the superior tarsal border to the OSASLA were measured. The upper–middle 1/3 of the superior tarsus was mattress-sutured to OSASLA with 5-0 absorbable sutures. We adjusted the position and tightness of suture for an 1-mm upper eyelid margin below the upper limbus (Fig. 1b). The skin incision was closed by a skin–OSASLA–skin suture to form a double eyelid.

### Success criteria

MRD1 ≥ 4 mm, the double eyelids formed well, the palpebral fissure was symmetrical, and the upper eyelids closed well.

### Statistical analysis

The distances from the superior tarsal border to the OSASLA and the upper eyelid elevation were tested. The measuring data were expressed as mean ± standard deviation, and the counting data were expressed as mean. Pearson’s correlation analysis was conducted, and *P* < 0.05 (two-sided) was considered statistically significant for all the statistical calculations. All statistical analyses were performed with PRISM 9.0 (GraphPad Software Inc., San Diego, CA, USA).

The study was approved by the Clinical Research Ethics Committee of our Hospital and performed in accordance with the tenets of the Declaration of Helsinki.

## Results

Among the 60 patients (92 eyes) with mild ptosis, the levator functions were 8 mm for 31 eyes, 9 mm for 23 eyes, 10 mm for 21 eyes, 11 mm for 10 eyes, and 12 mm for 7 eyes. Two patients (three eyes) underwent shortened LA, with a levator function of 8 mm for two eyes and 9 mm for one eye. The OSASLA sling was successfully performed in 58 (96.7%) patients. The distances from the OSASLA to the upper tarsus border were zero for two patients (three eyes), and shortening LA surgery was performed due to no feasibility of OSASLA sling. Some patients underwent partial skin excision to achieve a better double-eyelid shape.

**Fig. 2:**
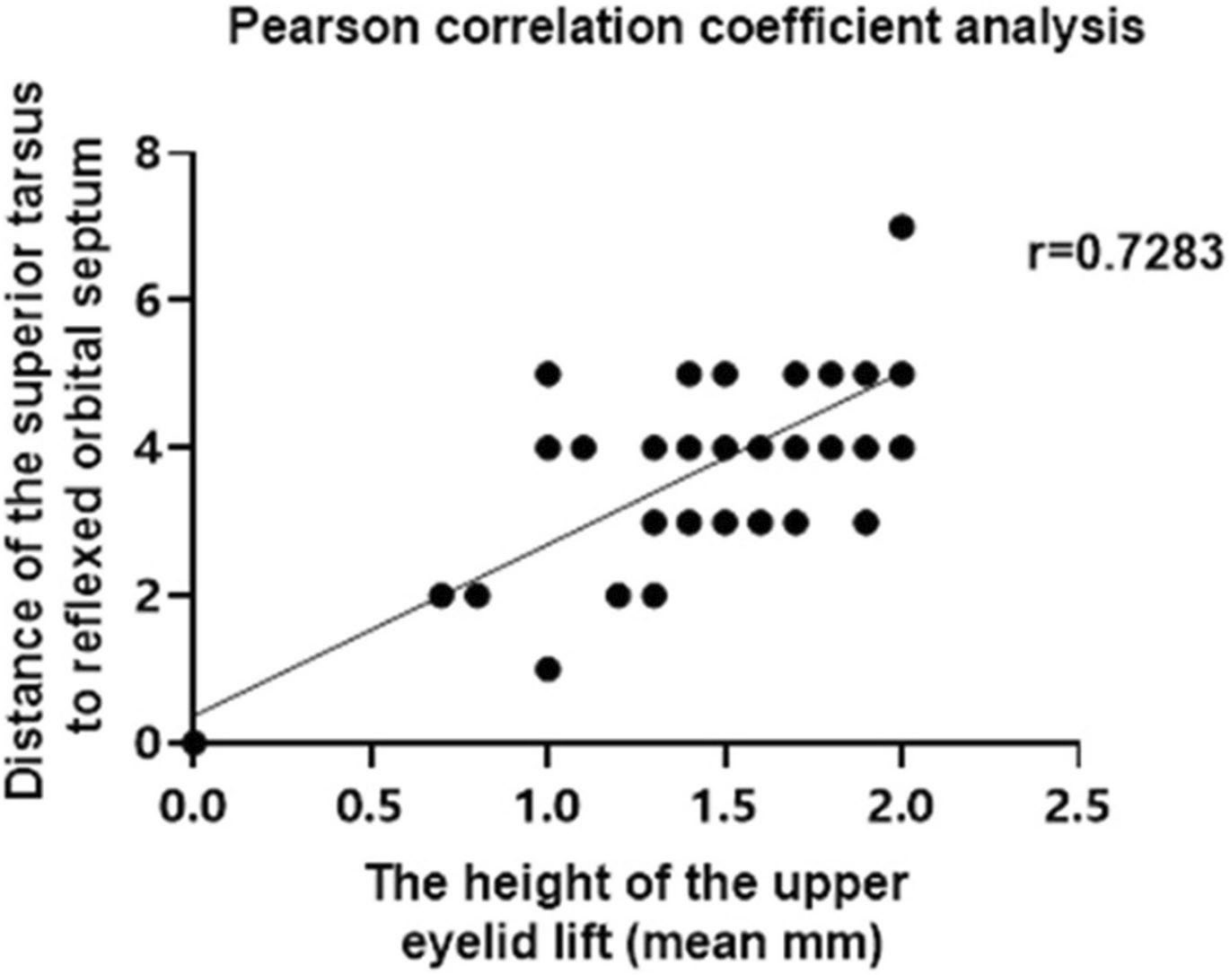
Correlation between the distance from the orbital septum attachment sites on the levator aponeurosis to the upper tarsal border and the height of the upper eyelid elevated

The average upper eyelid elevation value was 1.5 mm in 58 patients (89 eyes). The distances from OSASLA to superior tarsal borders ranged from 2 to 7 mm, and the elevated value of the upper eyelid was 0.7–2.2 mm. The lengths of the attachment site to the upper tarsus were 1–3 mm for 22 eyes, 4–5 mm for 65 eyes, and 7 mm for two eyes. The Pearson’s correlation coefficient analysis revealed a significantly positive correlation between the distance from the OSASLA to the upper tarsal border and the height of the upper eyelid elevated (*r* = 0.7283, *P* < 0.0001) (Fig. 2).

**Fig. 3:**
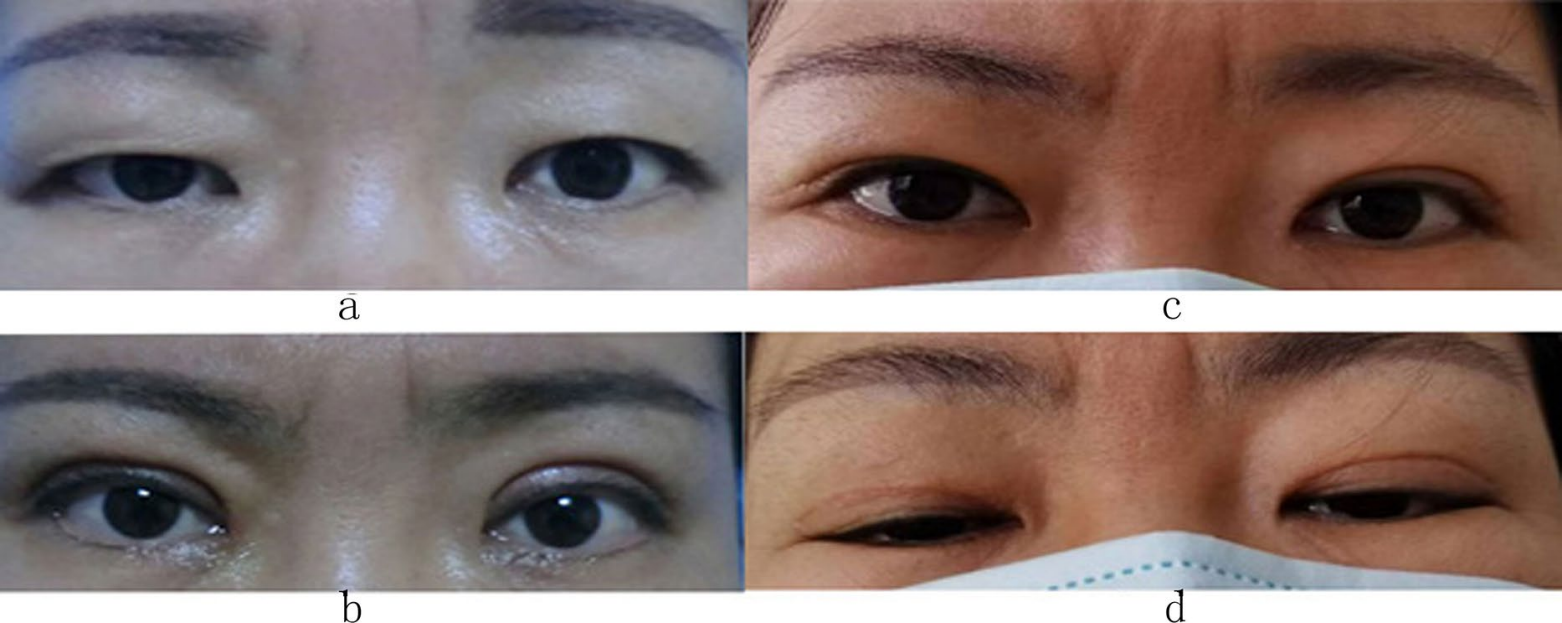
Mild blepharoptosis in the right eye (levator function 10 mm in the right eye and 12 mm in the left eye). **a** Before the operation. **b** One week after the operation. **c** Fourteen months after the operation. **d** Downward gaze 14 months after the operation

**Fig. 4:**
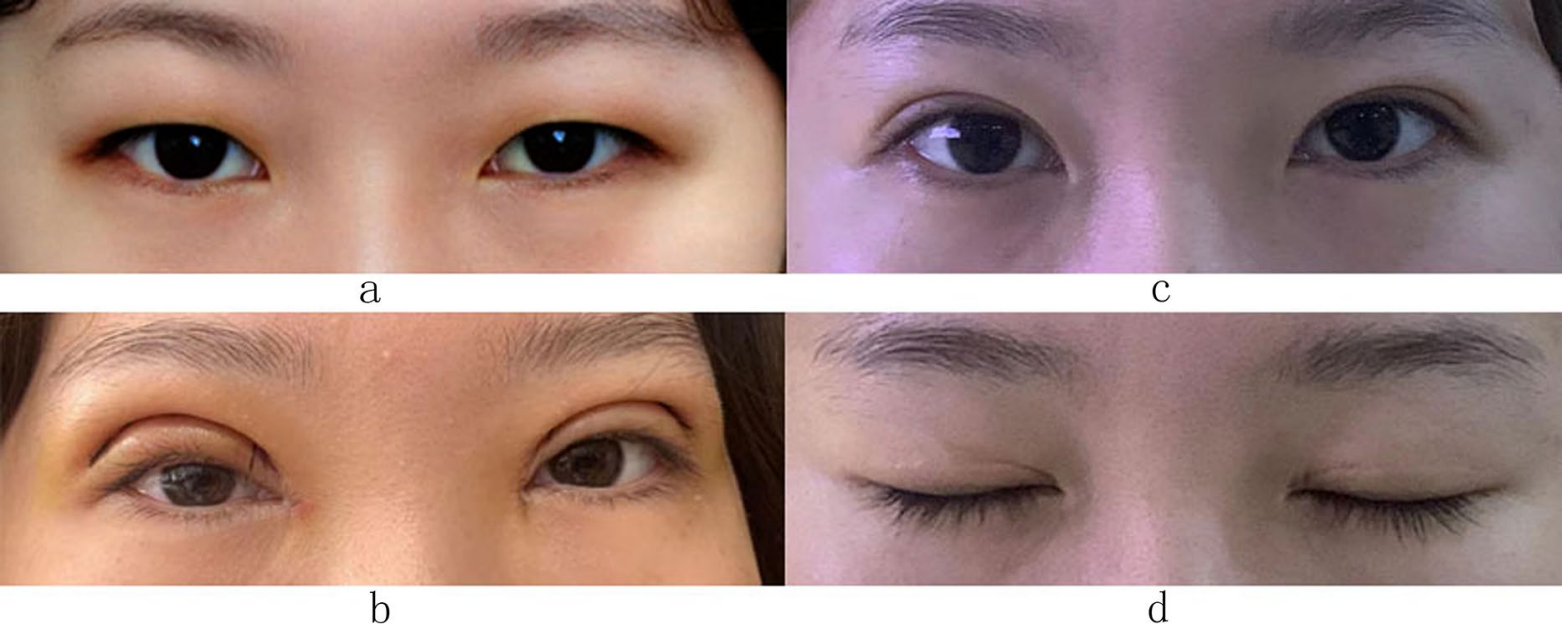
Mild blepharoptosis in both eyes (levator function 9 mm in both eyes). **a** Before the operation. **b** One week after the operation. **c** Eighteen months after the operation. **d** Eighteen months after the operation with eyes closed

After 6–18 months of follow-up, there were no apparent changes in the position of the upper eyelid margin and no upper eyelid lag (Figs. 3 and 4). The eyelid covered the palpebral fissure well, and there was no exposure keratitis or ptosis. The levator shortening was performed in two patients due to their moderate ptosis in the other eye. One patient was dissatisfied because the palpebral fissure height with the OSASLA sling was slightly smaller than the contralateral eye with shortened LA. The two eyes were asymmetrical when gazing downward. The other patient was satisfied with the appearance of the double eyelids after a second surgical repair for the narrow double eyelid 6 months after the surgery. The rest of the patients were all satisfied, achieving an overall satisfaction rate of 95%.

## Discussion

Many surgical options are available for mild ptosis, including tarsus–conjunctiva–Müller's muscle resection, shortening of LPS, and pleating of LPS [7, 8]. Tarsus–conjunctiva–Müller's muscle resection has been recognized because of the conjunctival approach and no scar on the skin. Gazit et al. [9] reported treating ptosis patients with levator function 5–10 mm, with an average MRD1 improvement of 2.8 mm, and the height and symmetry of bilateral eyelid fissures were good after the operation. Mangan et al. [10] reported Müller's muscle–conjunctival resection for contralateral ptosis after unilateral external levator advancement, achieving good results in 47 out of 52 cases. We previously performed the phenylephrine test on adult patients with 8–10-mm levator function. The positive ones were treated with Müller's muscle–conjunctival resection; they were easily overcorrected due to the influence of the side effect of phenylephrine during operation while undercorrected due to the disappearance of adrenalin factors after procedure. The operation was difficult to quantify and was abandoned. Furthermore, dissecting the Müller’s muscle during the operation can easily injure the levator aponeurosis, which may affect the postoperative results. Some surgeons consider resecting functional Müller's muscle unnecessary, while Jones and Mustarde advocate surgery aimed at preserving Müller’s muscle [11, 12].

The outcomes of LPS muscle shortening are consistent, yet the procedure is complex, and there are notable risks of postoperative upper eyelid drooping and exposure keratitis. Cates and Tyers [13] reported 100 congenital ptosis cases that underwent anterior levator resection, with 19% undercorrection and 7% overcorrection at 6 months. LPS shortening surgery requires dissection of the LA, which is complex and more invasive, requiring high demands on the surgeon and causing severe postoperative reactions. Furthermore, there is a higher likelihood of experiencing upper eyelid lag and incomplete closure of the palpebral fissure, which can result in exposure keratitis. In severer cases, the unusual white sclerotic can be seen when gazing downward. In our study, the postoperative results of the patients with mild ptosis were primarily associated with the distance between the OSASLA and the upper edge of the tarsus, rather than the levator function. Slinging of the upper tarsus to OSASLA is a simple operation that only involves opening the orbital septum without the need for a separate LA. Notably, our approach resulted in no postoperative upper eyelid drooping, exposure keratitis, or overcorrection.

While traditional pleating of LPS does not require a separate LA, it presents challenges in adjusting the position of the upper eyelid margin during the operation and is more prone to resulting in poor eyelid margin curvature. Additionally, there is an increased risk of postoperative upper eyelid position recession, primarily due to the gravitational impact of the overlapping LPS.

Kakizaki [14] reported that the distance from the superior tarsal plate border to the distal end of the anterior layer of the levator aponeurosis ranged from 0.7 to 8.6 mm in Asians. Similarly, we found that the distance from the OSASLA to the upper tarsus is 0–7 mm. There is a significantly positive correlation between the length from the OSASLA to the upper tarsus and the height of the upper eyelid elevated. The longer the distance from the attachment site to the upper tarsus, the greater the height of the upper eyelid elevated (with a maximum height of 2.2 mm). These findings indicate that OSASLA sling surgery is a suitable option for mild ptosis.

Postoperative eyelid swelling was minimal, thanks to the minimal damage incurred during the procedure. The attachment point remained fixed, resulting in a more natural upper lid margin curvature compared to other surgical methods. The cornea was well protected, and no postoperative exposure keratitis occurred. During follow-up examinations, we found that the eyelids of the affected eye that underwent OSASLA sling surgery demonstrated well closure, with no apparent upper eyelid lag when looking downward. The upper eyelid exhibited flexible opening and closing, and the esthetic outcome was similar to that of double-eyelid surgery, which the patients easily accept. Notably, there was no upper eyelid retraction in patients followed up for 6–18 months.

In summary, the OSASLA sling procedure for mild congenital ptosis is more effective, easier to perform, and minimally invasive. It represents a novel surgical technique to correct mild congenital ptosis in Asian patients with good levator function. It is essential to mention that this technique has not been studied in the context of acquired blepharoptosis and other associated abnormalities. Additionally, due to the limited number of patients in our study, its suitability for occidental eyelids warrants further investigation.

## Data Availability

The datasets during and/or analyzed during the current study are available from the corresponding author upon reasonable request.
